# The Interaction of the Flavonoid Fisetin with Human Glutathione Transferase A1-1

**DOI:** 10.3390/metabo11030190

**Published:** 2021-03-23

**Authors:** Mohammed Hamed Alqarni, Ahmed Ibrahim Foudah, Magdy Mohamed Muharram, Nikolaos E. Labrou

**Affiliations:** 1 Department of Pharmacognosy, College of Pharmacy, Prince Sattam Bin Abdulaziz University, Alkharj 11942, Saudi Arabia; a.foudah@psau.edu.sa; 2 Department of Pharmaceutics, College of Pharmacy, Prince Sattam Bin Abdulaziz University, Alkharj 11942, Saudi Arabia; m.moharm@psau.edu.sa; 3 Department of Microbiology, College of Science, Al-Azhar University, Nasr City, Cairo 11884, Egypt; 4 Laboratory of Enzyme Technology, Department of Biotechnology, School of Food, Biotechnology and Development, Agricultural University of Athens, 75 Iera Odos Street, GR-11855 Athens, Greece

**Keywords:** chemosensitization, glutathione transferase, fesitin, multidrug resistance, senescence

## Abstract

Glutathione transferases (GSTs) are a family of Phase II detoxification enzymes that are involved in the development of the multidrug resistance (MDR) mechanism in cancer cells and therefore affect the clinical outcome of cancer chemotherapy. The discovery of nontoxic natural compounds as inhibitors for GSTs is a promising approach for chemosensitizing and reversing MDR. Fisetin (7,3′,4′-flavon-3-ol) is a plant flavonol present in many plants and fruits. In the present work, the interaction of fisetin with human glutathione transferase A1-1 (hGSTA1-1) was investigated. Kinetic analysis revealed that fisetin is a reversible inhibitor for hGSTA1-1 with IC_50_ 1.2 ± 0.1 μΜ. It functions as a mixed-type inhibitor toward glutathione (GSH) and as a noncompetitive inhibitor toward the electrophile substrate 1-chloro-2,4-dinitrobenzene (CDNB). In silico molecular modeling and docking predicted that fisetin binds at a distinct location, in the solvent channel of the enzyme, and occupies the entrance of the substrate-binding sites. Treatment of proliferating human epithelial colorectal adenocarcinoma cells (CaCo-2) with fisetin causes a reduction in the expression of hGSTA1-1 at the mRNA and protein levels. In addition, fisetin inhibits GST activity in CaCo-2 cell crude extract with an IC_50_ (2.5 ± 0.1 μΜ), comparable to that measured using purified recombinant hGSTA1-1. These actions of fisetin can provide a synergistic role toward the suppression and chemosensitization of cancer cells. The results of the present study provide insights into the development of safe and effective GST-targeted cancer chemosensitizers.

## 1. Introduction

Glutathione transferases (GSTs), through their catalytic and noncatalytic roles, are able to perform multiple functions [1,2,3,4,5,6]. The main catalytic role of GSTs relies on their ability to provide detoxification to the cell from various hydrophobic, electrophilic and toxic molecules, through their conjugation with reduced glutathione (GSH) [4,5,6,7]. Cytoplasmic GSTs are divided into different classes in mammals, plants, insects, bacteria and fungi [4,5]. The human cytosolic GST gene family encodes sixteen genes, grouped into six classes: alpha (GSTA), mu (GSTM), omega (GSTO), pi (GSTP), theta (GSTT) and zeta (GSTZ). This classification is based on nucleotide and amino acid sequences, tertiary and quaternary structures, substrate specificity and immune cross-reactivity [4,5]. GSTs that belong to the same class show more than 40% similarity in terms of amino acid sequence, while GSTs that belong to different classes share less than 25% homology. Cytoplasmic GSTs consist of two subunits and can be either homodimers, encoded by one gene, or heterodimers, encoded by different genes. Each subunit consists of 200–250 amino acid residues, and their molecular mass ranges between 20 and 28 kDa. A single active site is located in each subunit, which is composed of two discrete subsites: the GSH-binding site (G-site) in its N-terminal domain and a hydrophobic substrate-binding site (H-site) in the carboxy terminal region [4,5].

Among the electrophilic molecules that are detoxified by GSTs are various alkylating drugs that are currently being used in the treatment of cancer. This detoxifying role, in combination with the overexpression of GSTs in several types of cancer cells, contributes to the resistance of cancer cells to alkylating agents [1,7,8,9,10,11,12,13,14,15,16,17]. This is achieved either by attenuating the efficacy of anticancer drugs or by inhibiting the mitogen-activated kinase pathway (MAP). Different GST isoenzymes have been shown to be involved in these functions, such as hGSTA1-1 [10,16,17], hGSTP1-1 [2,8,14], hGSTO1-1 [12], hGSTM1-1 and hGSTM3-3 [15]. These actions are of great significance because they may compromise the efficacy and therefore the successful outcome of therapeutic approaches [3,10,13]. To this end, one of the strategies that are being investigated to address this problem relies on the use of specific molecules that are able to inhibit the function of GSTs [10,11]. Several potent inhibitors have been developed over the last years, which are classified into two groups: those that bind to the G-site and those that bind to the H-site. Numerous compounds, natural or synthetic, with different degrees of efficacy and inhibition potency, have been reported [18,19,20,21,22,23,24].

**Figure 1:**
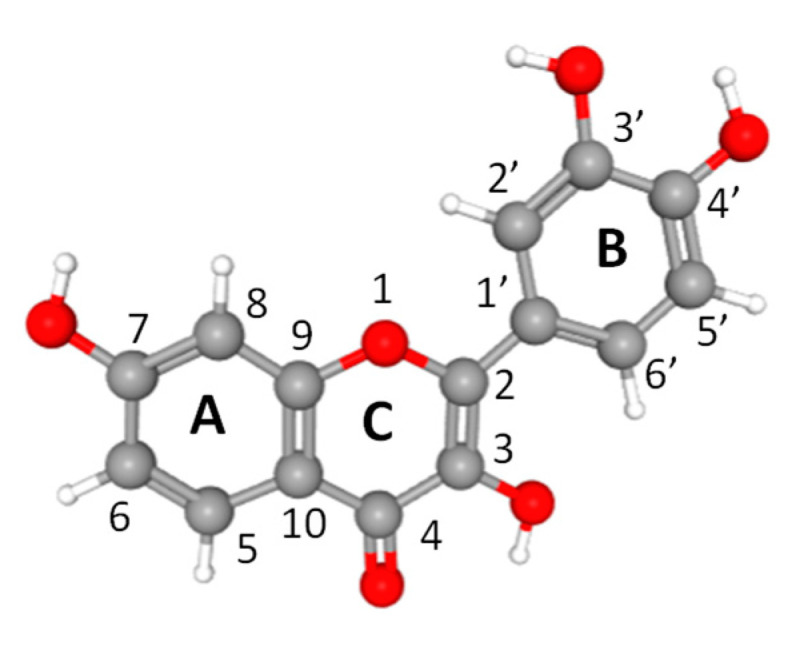
Ball-and-stick model and atom numbering of fisetin. Atoms are colored according to their type: oxygen, red; carbon, gray; hydrogen, white.

Flavonoids are widely consumed by humans and are found in plants, mainly fruits and vegetables [24,25,26]. They show high antioxidant and free radical scavenging effects, and recent studies have shown that flavonoids exhibit a wide range of activities in several diseases, including neurodegenerative diseases, stroke, depression, diabetes and cancer [24,25,26,27,28,29]. Fisetin (7,3′,4′-flavon-3-ol) is a plant flavonol that belongs to the flavonoid group of polyphenols (Figure 1). Recent studies have reported its involvement in multiple signaling pathways and established its wide range of bioactivities, such as anti-inflammatory, anti-invasive, antitumorigenic, antiangiogenic, antidiabetic, cardioprotective and neuroprotective activities [24,25,26,27,28,29]. It has also been reported that fisetin displays a senotherapeutic function and is able to extend health and lifespan [30,31]. Senotherapeutics are drugs that can affect senescent cells and interfere with their proaging impacts. Senotherapeutic drugs function either by killing senescent cells (senolytic drugs) or by inhibiting their function (senostatic drugs) [31].

Fisetin has been reported to exert pleiotropic effects in different diseases and proteins both in vitro and in vivo [30,32]. Different biological events and protein targets can be controlled by fisetin. For example, several regulatory proteins such as antiapoptotic and proapoptotic proteins, cyclin-dependent kinases (CDKs), cyclins, matrix metalloproteinases (MMPs) and growth factors have been shown to be modulated by fisetin [30,31].

The present work aims to investigate the interaction of fisetin with hGSTA1-1. Considering that fisetin displays senotherapeutic function and taking into account that hGSTA1-1 is involved in anticancer drug resistance [16,17], the present work was undertaken in order to investigate whether fisetin affects the catalytic activity and expression of hGSTA1-1. The results of the present work provide new insights into the drug design effort toward GSTs. The outcome of the study can facilitate a more rational development of safe and effective GST-targeted chemosensitizers for reversing MDR.

## 2. Results and Discussion

### 2.1. The Inhibition of Recombinant hGSTA1-1 by Fisetin

**Figure 2:**
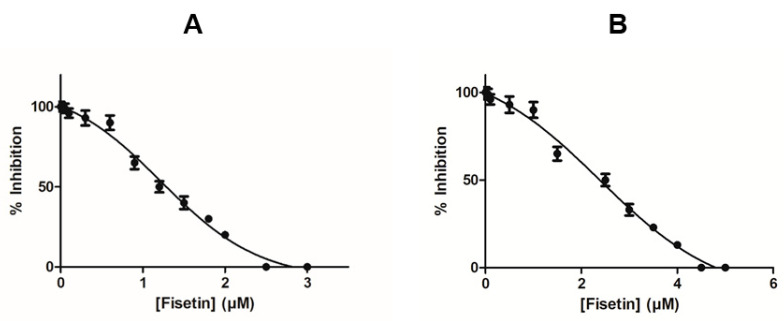
Concentration–response curves for the determination of IC_50_ values for fisetin. The graph was produced using GraphPad Prism 5. (**A**) Concentration–response curve using recombinant hGSTA1-1. (**B**) Concentration–response curve using CaCo-2 cell-free crude extract.

The effects of fisetin on the activity of hGSTA1-1 were assessed using CDNB and GSH as substrates. Figure 2A shows the dependence of the enzyme’s conjugation activity on fisetin concentration. The IC_50_ value for fisetin was determined as 1.2 ± 0.1 μΜ. Fisetin appears to be a strong inhibitor for hGSTA1-1, compared to other synthetic or natural inhibitors that have been studied over the last years [18,19,20,21,23,32,33]. For example, the IC_50_ values for colchicine [23] or synthetic 2-(pyrrolesulfonylmethyl)-N-arylimines, benzophenones and their carbonyl N-analog ranged between 22 and 71 μΜ [32,33]. However, they fall within the range found for xanthone derivatives [19].

**Figure 3:**
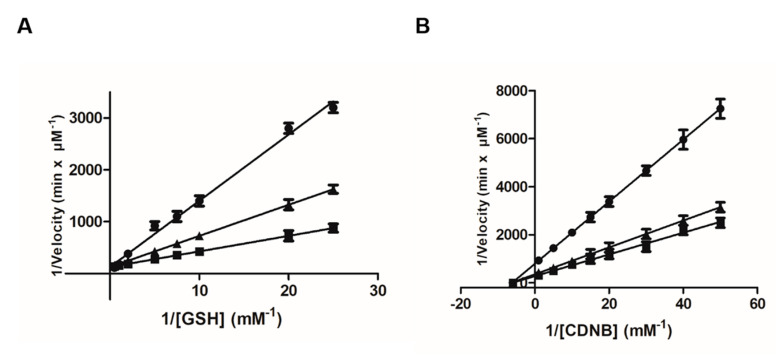
Lineweaver–Burk plots for the inhibition of hGSTA1-1 by fisetin. (**A**) Inhibition of hGSTA1-1 by fisetin [(0 μM (■), 0.5 μM (▲), 2.5 μM (●)] using the concentration of the CDNB constant, and the concentration of GSH was varied (0.04–2.0 mM). (**Β**) Inhibition of hGSTA1-1 by fisetin [(0 μM (■), 1 μM (▲), 3.5 μM (●)] using the concentration of GSH constant, and the concentration of CDNB was varied (0.0375–0.675 mM).

Steady-state kinetics inhibition analysis was used for determining fisetin’s potency (*K_i_*), type of inhibition and the impact of its concentration on the steady-state parameters (*K_M_* and *k_cat_*) of the enzymatic reaction. Figure 3 shows the Lineweaver–Burk plots of hGSTA1-1 kinetics in the presence and absence of different concentrations of fisetin. Fisetin acts as a mixed-type inhibitor toward GSH and as a noncompetitive inhibitor toward CDNB, with *K_i_* values of 0.5 ± 0.1 μM and 1.1 ± 0.03 μΜ, respectively. Similar types of inhibition have also been found by other synthetic inhibitors such as pyrrole, xanthone and benzophenone analogs, with different structures and potency [32,33].

### 2.2. The Effect of pH, Temperature and Viscosity on IC_50_

**Figure 4:**
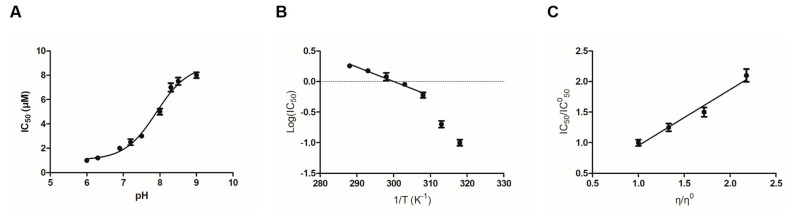
Dependence of IC_50_ (μΜ) on pH (**A**), temperature (**B**) and viscosity (**C**).

The effect of pH on the inhibition potency (IC_50_) of fisetin was evaluated to study the enzyme’s ionizable group(s) that contribute to its binding. Figure 4A illustrates the dependence of pH (6.0–9.0) on IC_50_. A sigmoid curve was observed, suggesting that the binding is highly dependent on the acid/base properties of a specific amino acid side chain that interacts directly with fisetin. The transition observed corresponds to pK_a_ 7.9 ± 0.2. Although, based exclusively on pK_a_ value, we cannot decide unequivocally on the identity of the ionizable groups, the inflection point at pH 7.9 indicates that a Lys, Cys or Tyr residue presumably contributes directly to fisetin binding. This residue is presumably the main structural determinant conferring tight binding. A similar profile has been observed by studying the pH dependence of the kinetic parameters of alpha-class GSTs [34,35].

The effect of temperature on the inhibition potency is shown in Figure 4B, in which the Arrhenius plot of the logarithm of IC_50_ against the reciprocal of the absolute temperature gave a line. The formation of the enzyme–fisetin complex is exothermic, and the effect of temperature is approximately linear up to 35 °C, where a break occurs with a steepening of the slope. The cause of two phases in the plot is obscure; the most tenable explanation appears to be that some change in conformation takes place at this temperature, altering the affinity of the enzyme for fisetin.

Next, we examined the effect of viscosity on IC_50_ to assess whether the binding of the inhibitor to hGSTA1-1 is controlled by a diffusion-controlled structural transition of the protein. The dependence of IC_50_ by increasing the medium viscosity by glycerol indicates the influence of diffusion on binding [36,37]. In relation to Kramers’ theory, enzymes that undergo conformation changes during the binding of an inhibitor should be affected by the viscosity of the medium [36,37]. In a diffusion-dependent binding of the inhibitor, the inhibition constant is affected by the friction of the solvent with the enzyme because friction affects the free energy needed to reach the transition state. In turn, friction is a function of viscosity η [36,37]. A plot of the relative IC_50_ (IC_50_/IC°_50_) against the relative viscosity (η/η°) (IC°_50_ and η° were determined in the absence of glycerol) should be linear when a structural transition is limited by a strictly diffusional barrier. As shown in Figure 4C, the relative IC_50_ for the enzyme–fisetin complex shows a linear dependence on the relative viscosity with a slope very close to unity (0.9165 ± 0.1105).

### 2.3. The Interaction of hGSTA1-1 and Fisetin by In Silico Molecular Docking

**Figure 5:**
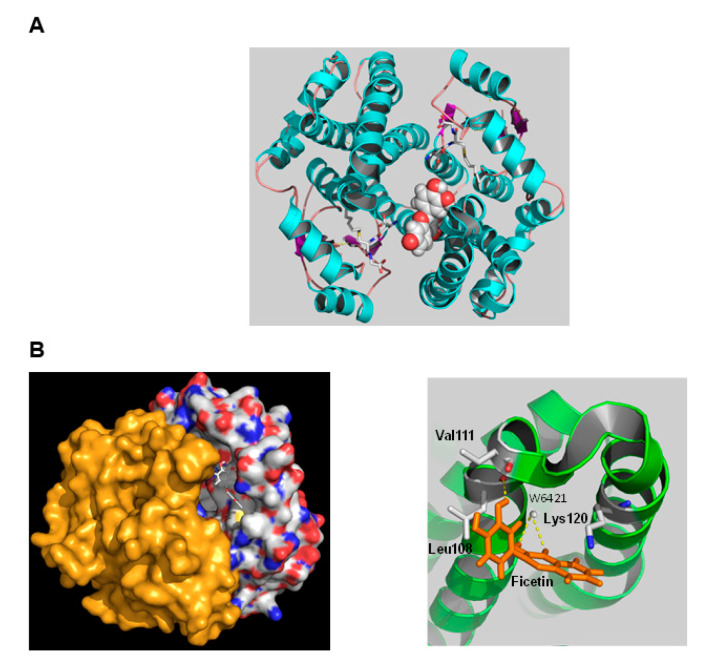
(**A**) Predicted favorable binding mode of fisetin in hGSTA1-1. Fisetin is shown as spheres. The bound inhibitor S-hexyl-GSH, overlapping both the G- and H-sites, is shown in a stick representation and colored according to the atom type. (**B**) Fisetin is shown in a stick representation, and the enzyme subunits are shown as surface (left). The fisetin-bound subunit is colored according to the atom type. Important side chains that contribute to interaction are shown in a stick representation, colored according to the atom type and labeled (right).

The interaction of fisetin with hGSTA1-1 was also studied by in silico molecular docking [38]. The most favorable binding mode of fisetin with hGSTA1-1 (deltaG = −7.21, FullFitness = −2002.3) is shown in Figure 5. The binding site of fisetin is located at a distinct position at the solvent channel and occupies the entrance of the substrate-binding site. Fisetin interacts with residues from helices A4 and A5. Analysis of the putative binding site indicates that the interaction of fisetin with the enzyme is accomplished by both polar and nonpolar interactions. Important residues that contribute to binding involve those from the highly hydrophobic segment 106-ILLPV-111, such as Val111 and Leu108. The 3′-hydroxyl group of fisetin is hydrogen bonded with the main chain carbonyl group of Leu108. Two other hydrogen bonds are formed between the 3-hydroxyl and 4-carbonyl groups of fisetin with a water molecule (W6421). The side chain ε-amino group of Lys120 is positioned above the aromatic A-ring of fisetin, allowing the formation of amino–aromatic interaction. Presumably, pK_a_ 7.9, observed in the IC_50_ vs. pH profile (Figure 4A), corresponds to Lys120, which contributes to fisetin binding through this amino–aromatic interaction.

The predicted location of the fisetin-binding site appears to be in agreement with the results obtained by kinetic inhibition analysis and confirms the observed noncompetitive type and the mixed-type of inhibition. Given the distance between the fisetin-binding site and the G- and H-sites, it is plausible that the enzyme can simultaneously accommodate both fisetin and substrates (GSH/CDNB). The binding to fisetin at the entrance of the substrate-binding site may cause a significant impact on the dynamics of the subunit interface, the accessibility of the solvent channel by the substrates, the binding of GSH and CDNB and the release of the reaction product.

### 2.4. The Effect of Fisetin on hGSTA1-1 Expression and Inhibition in Caco-2 Cells

**Figure 6:**
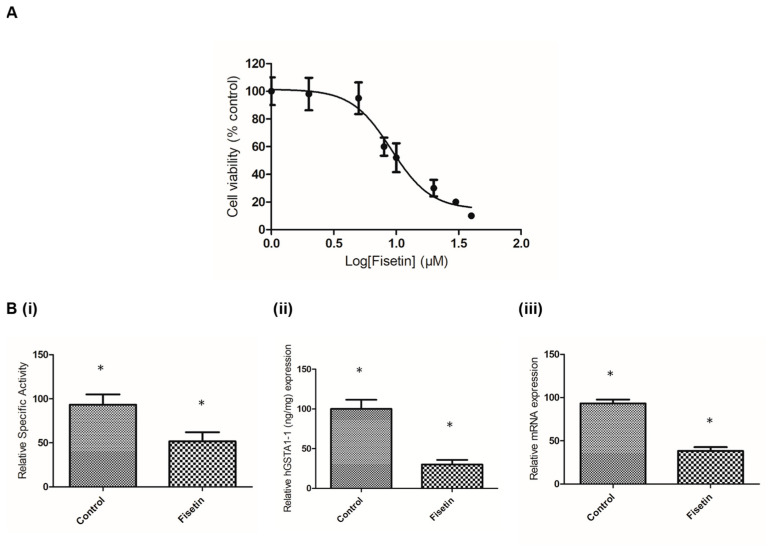
(**A**) Effect of fisetin concentration on the viability of proliferating CaCo-2 cells after a 24 h treatment. Cell viability was assayed using the neutral red uptake assay. Data are presented as a percentage of the respective control (=100%). Results are presented as the mean ± S.D. of 3 independent experiments. (**B**) Relative enzyme-specific activity (**i**) and accumulation of hGSTA gene transcripts (**ii**) under fisetin (1 µM) treatment in proliferating CaCo-2 cells after 24 h. Total RNA was isolated, reverse transcribed to cDNA and subjected to real-time quantitative PCR using gene-specific primers. Relative mRNA level was calculated with respect to the respective expression level in the control samples after normalization with the levels of the glyceraldehyde 3-phosphate dehydrogenase (GAPDH) and beta-2 microglobulin (B2M) transcripts. Results are presented as the mean ± SD of 3 independent experiments. The asterisks indicate statistical significance at the 0.05 level.

Proliferating CaCo-2 cells were exposed to fisetin within the concentration range of 0–100 µM, and cell viability was measured using the neutral red uptake (NRU) assay. Figure 6A depicts the concentration-dependent decrease in the cell viability after 24 h. The half-maximal inhibitory concentration value was measured as 9.1 ± 3.2 µM. GST activity was measured in CaCo-2 cell-free crude extract that was treated with or without 1 μΜ of fisetin after 24 h. The results of the activity assays show that fisetin treatment lowered the expression of total GST activity by about 61.5 ± 4% (Figure 6B). In addition, the levels of hGSTA1-1 were also determined using ELISA assay. The results confirmed that fisetin reduced the expression of the enzyme (Figure 6B). The ability of fisetin to function as an inhibitor toward hGSTA1-1 was also assessed using CaCo-2 cell-free crude extract. In agreement with the results obtained using purified recombinant hGSTA1-1 (Figure 2A), a similar concentration-dependent decrease in enzyme activity was found. The IC_50_ value was measured 2.5 ± 0.1 µM (Figure 2B), suggesting that fisetin retained its ability to act as a potent inhibitor toward the GST activity in CaCo-2 crude extract.

The downregulation of GST was also observed at the mRNA level (mRNA for GSTA1/2), as measured using RT-qPCR, compared with the untreated CaCo-2 cells. The results of both activity assays and RT-qPCR are illustrated in Figure 6B. Obviously, the reduction in hGSTA1-1 expression in both protein and mRNA levels in CaCo-2 cells appears to be linked to fisetin treatment.

GSTA1-1 is highly expressed in many non-small-cell lung cancer cell lines and plays a crucial role in the development of anticancer drug resistance. Its downregulation leads to suppression of tumor growth and the induction of cell apoptosis [39,40]. Therefore, the downregulation of the GSTA1-1 mRNA expression caused by fisetin in proliferating CaCo-2 cells, observed in the present work, suggests a synergistic function of fisetin regarding the suppression and chemosensitization of cancer cells.

In noncancerous cells and tissues, the upregulation of GSTs expression is considered beneficial to the cells because GST protects cells against potentially harmful electrophiles [41]. Krajka-Kuzniak et al. reported that in THLE-2 hepatocytes, a significant increase in the protein expression levels of GSTP and GSTT isoenzymes was found upon treatment with the chalcone xanthohumol [42]. Clinical studies have shown that the administration of xanthohumol to human volunteers resulted in enhanced levels of GSTA in plasma, while the levels of GSTP remained unaltered [43]. In another work, Dietz et al. observed the significantly increased enzymatic activity of GST in the liver after administration of a hop extract in rats [44].

Senescence is considered a tumor-suppressive process. It functions by preventing cancer cell proliferation and suppressing malignant progression from premalignant to malignant disease [45,46]. Currently, drugs that are able to kill senescent cells (senolytic drugs) or inhibit their function (senostatic drugs) are under intense research, aiming to establish whether their clinical use can increase the efficacy of cancer therapies and improve resilience to cancer treatments. Fisetin is regarded as a senolytic natural product and. together with quercetin and navitoclax, is under preclinical and clinical investigation for investigating their potential role in cancer treatments [31]. Taking into account that fisetin displays high senolytic activity as well as high inhibition potency toward hGSTA1-1, a synergistic role for fisetin can be proposed, providing both senolytic and chemosensitization function to cancer cells.

## 3. Materials and Methods

### 3.1. Materials

Reduced glutathione (GSH), 1-chloro-2,4-dinitrobenzene (CDNB), bovine serum albumin (BSA), fisetin and ampicillin were purchased from Sigma–Aldrich (St. Louis, MO, USA). CaCo-2 cell line (human epithelial colorectal adenocarcinoma) was purchased from ATCC.

### 3.2. Heterologous Expression and Purification of Recombinant hGSTA1-1

The hGSTA1-1was expressed in *E. coli* BL21 (DE3) and purified by affinity chromatography on immobilized GSH as described before [47]. Protein concentration was determined by the Bradford method, using BSA as standard.

### 3.3. Assay of GST Activity and Inhibition Analysis by Fisetin

GST assays were achieved using the 1-chloro-2,4-dinitrobenzene (CDNB)/GSH system as described before [47]. For the determination of IC_50_ of fisetin for hGSTA1-1, the reaction mixture contained different concentrations of fisetin (0.02–3 μΜ). The IC_50_ values were determined from plots of the percentage of remaining enzyme activity vs. fisetin concentration. The software GraphPad Prism 5 Software, (San Diego, CA, USA) was used for the analysis.

### 3.4. Kinetic Measurements

Steady-state kinetic analysis of the CDNB/GSH conjugation reaction of hGSTA1-1 was achieved using 0.0375–0.675 mM CDNB in the presence of 2.5 mM GSH in 0.1 M sodium phosphate buffer, pH 6.5 (37 °C). Alternatively, the dependence of the initial rates of the enzymatic reaction on GSH concentration was accomplished using 0.04–2.0 mM GSH and 1.5 mM CDNB. The kinetic data were analyzed by nonlinear regression analysis using the computer program GraphPad Prism 5 (San Diego, CA, USA). All assays were carried out in triplicate, and blanks were used to account for spontaneous CDNB/GSH conjugation reaction.

### 3.5. The Dependence of IC_50_ on Viscosity, pH and Temperature

The effect of viscosity on IC_50_ was studied in 0.1 M potassium phosphate buffer, pH 6.5, containing variable glycerol concentrations (0–30% *v*/*v*). Viscosity values were calculated as described in Wolf et al. [48]. Analysis of pH dependence of IC_50_ was achieved in 0.1 M potassium phosphate buffer (pH range: 6.0–8.0) and Tris/HCl buffer (pH range: 8.0–9.0). The effect of temperature (10–40 °C) on IC_50_ was carried out in 0.1 M potassium phosphate buffer pH 6.5. Under all assay conditions, the enzyme remained stable.

### 3.6. Molecular Modeling

The putative binding site of fisetin was identified by molecular docking using the SwissDock program and EADock DSS software [38,49]. The structure of hGSTA1-1 with PDB code 1K3L was used. For the evaluation of the docking results, CHARMM energies were measured, and the ligand-binding modes were evaluated using FACTS and clustered [38,49]. The program PyMOL (http://www.pymol.org/) was used for modeling and visualizing 3D structures.

### 3.7. Cultivation of the CaCo-2 Cell Line

Eagle’s Minimum Essential Medium (EMEM) was used for culturing CaCo-2 cells in Petri dishes for 3 days before treatment. The EMEM was supplemented with 10% (*v*/*v*) heat-inactivated fetal bovine serum, 1% (*v*/*v*) nonessential amino acids, 1% (*v*/*v*) glutamine and 0.5% penicillin/streptomycin. CaCo-2 cells were grown in a humidified atmosphere containing 5% (*v*/*v*) CO_2_ at 37 °C to obtain proliferating cells.

### 3.8. Treatment of CaCo-2 Cells with Fisetin and GST Activity Measurements

Proliferating CaCo-2 cells were treated with fisetin (1 µM). The cells were incubated for 24 h and harvested into a 0.1 M sodium phosphate buffer (pH 7.4) or used for RNA extraction. Before harvesting, CaCo-2 cells were rinsed with phosphate-buffered saline (PBS). Cell viability was assayed using the neutral red uptake assay [50]. Cytosolic extract of CaCo-2 cells was obtained by homogenization using sonication (200 W, 26 kHz, 30 s) in 0.1 M sodium phosphate buffer pH 7.4 or 0.1 Tris/HCl buffer pH 7.4. The lysed cells were centrifuged (10,000× *g*, 4 °C), and the supernatant was collected and used for GST activity determination (U/mg). The levels of hGSTA1-1 (ng/mL) were also determined using an ELISA kit (Abcam, UK) according to manufacturer instructions. The cytosolic GST activity was assayed using the CDNB/GSH conjugation reaction at 37 °C, according to Axarli et al. [47].

### 3.9. RNA Extraction and RT-qPCR

The total RNA isolation kit (Promega, Madison, WI, USA) was used for RNA isolation. The samples were treated with DNase I (37 °C, 45 min). First-strand synthesis was achieved using SuperScript II and random hexanucleotides. GST transcripts were amplified using gene-specific primers (Supplementary Table S1). PCR amplification cycles included: a first denaturation cycle of 10 min at 95 °C, followed by 40 cycles composed of 15 s at 95 °C and 1 min at 60 °C. The relative expression levels of the target gene were calculated using the 2^−ΔΔCt^ method [51]. All assays were carried out in three biological repeats. The geometric mean of beta-2 microglobulin (B2M) and glyceraldehyde 3-phosphate dehydrogenase (GAPDH) was used for normalizing the expression level of the target enzyme. The results were expressed as the fold change relative to the control.

### 3.10. Statistical Analysis

The presented data were obtained from three independent measurements. GraphPad Prism 5 (GraphPad Software, San Diego, CA, USA) was used for data analysis and evaluation.

## 4. Conclusions

Research over the past two decades has shed light on the role of GSTs as important determinants of therapeutic response to chemotherapy. GSTs are significant targets for drug design because the inhibition of their catalytic activity can provide a direct and well-defined effect on drug metabolic pathways and cell signaling. The results of the present work showed that fisetin is a strong inhibitor toward recombinant and native hGSTA1-1. In addition, fisetin negatively affects the expression of hGSTA1-1 at mRNA and protein levels in CaCo-2. The outcome of the study can facilitate a more rational development of safe and effective GST-targeted cancer chemosensitizers.

## Data Availability

Data are available upon request.

## Supplementary Materials

The following are available online at https://www.mdpi.com/2218-1989/11/3/190/s1, Table S1: List of the primers used for RT-qPCR analysis of the selected genes.
