# Supplementary material for: The Interaction of the Flavonoid Fisetin with Human Glutathione Transferase A1-1
Source: Metabolites. 2021 Mar 23;11(3):190. doi: 10.3390/metabo11030190 (PMC8004991; doi:10.3390/metabo11030190)
Supplement: Supplementary file 1 [file metabolites-11-00190-s001.pdf]

Supplementary Table S1

List of the primers used for RT-qPCR analysis of the selected genes.

Gene	Forward Primer	Reverse Primer
Target gene		
GSTA1/2	GTGCAGACCAGAGCCATTC	TCACCCAAATCTGCTATACCTTC
Reference genes		
B2M	TGCTGTCTCCATGTTTGATGTATC	TCTCTGCTCCCCACCTCTAAG
GAPDH	GAGTCCACTGGCGTCTTCAC	GAGGCATTGCTGATGATCTTGAG
